# Colon-available raspberry polyphenols exhibit anti-cancer effects on *in vitro *models of colon cancer

**DOI:** 10.1186/1477-3163-6-4

**Published:** 2007-04-18

**Authors:** Emma M Coates, Gina Popa, Chris IR Gill, Mark J McCann, Gordon J McDougall, Derek Stewart, Ian Rowland

**Affiliations:** 1 Northern Ireland Centre for Food and Health (NICHE), University of Ulster (Coleraine), Cromore Road, Coleraine, Co. Londonderry BT52 1SA, Northern Ireland, United Kingdom; 2 Scottish Crop Research Institute (SCRI) Quality, Health and Nutrition Programme, Mylnefield, Invergowrie, Dundee DD2 5DA, Scotland, United Kingdom

## Abstract

**Background:**

There is a probable association between consumption of fruit and vegetables and reduced risk of cancer, particularly cancer of the digestive tract. This anti-cancer activity has been attributed in part to anti-oxidants present in these foods. Raspberries in particular are a rich source of the anti-oxidant compounds, such as polyphenols, anthocyanins and ellagitannins.

**Methods:**

A "colon-available" raspberry extract (CARE) was prepared that contained phytochemicals surviving a digestion procedure that mimicked the physiochemical conditions of the upper gastrointestinal tract. The polyphenolic-rich extract was assessed for anti-cancer properties in a series of *in vitro *systems that model important stages of colon carcinogenesis, initiation, promotion and invasion.

**Results:**

The phytochemical composition of CARE was monitored using liquid chromatography mass spectrometry. The colon-available raspberry extract was reduced in anthocyanins and ellagitannins compared to the original raspberry juice but enriched in other polyphenols and polyphenol breakdown products that were more stable to gastrointestinal digestion. Initiation – CARE caused significant protective effects against DNA damage induced by hydrogen peroxide in HT29 colon cancer cells measured using single cell microgelelectrophoresis. Promotion – CARE significantly decreased the population of HT29 cells in the G_1 _phase of the cell cycle, effectively reducing the number of cells entering the cell cycle. However, CARE had no effect on epithelial integrity (barrier function) assessed by recording the trans-epithelial resistance (TER) of CACO-2 cell monolayers. Invasion – CARE caused significant inhibition of HT115 colon cancer cell invasion using the matrigel invasion assay.

**Conclusion:**

The results indicate that raspberry phytochemicals likely to reach the colon are capable of inhibiting several important stages in colon carcinogenesis *in vitro*.

## Background

Epidemiological studies have consistently shown association between the consumption of fruit and vegetables and a reduced risk of human pathologies, such as cardiovascular disease and cancer [1-3]. Cancers of the digestive tract, in particular colorectal cancer (CRC), are amongst those most responsive to dietary modification. Epidemiological studies have shown that approximately 75 % of all sporadic cases of CRC are directly influenced by diet [4], and that dietary modification is a feasible strategy for reducing CRC risk [5].

There are many stages involved in the development of cancer and those involved in CRC development have been well studied [6,7]. The main events in colon cancer development can be classified as initiation, promotion and metastasis. Under certain conditions, the overproduction of reactive oxygen species can lead to free radical induced damage to DNA [8]. Left unrepaired by the endogenous DNA repair mechanisms [9] this damage could be incorporated as a permanent sequence change, potentially initiating the multi-step pathways involved in carcinogenesis [2,10]. Normal cell growth and proliferation are regulated by tumour suppressor genes and proto-oncogenes, which control cell cycle and apoptosis. However, several tumour suppressor genes are known to be inactivated with the parallel activation of key oncogenes via mutation, which can result in upregulated and uncontrolled proliferation of cancer cells, allowing colon cancer to progress [7,11].

The protective effect of fruits and vegetables may be due to the biological activities of dietary fibre, vitamins, minerals and/or phytochemicals [12,13]. Phytochemicals can be defined as bioactive non-nutrient components of fruits and vegetables and are split into groups such as carotenoids, polyphenols, alkaloids and other nitrogen-containing compounds [14]. The chemopreventive nature of certain phytochemicals may arise because of their antioxidant activity or, more precisely, their ability to inactivate reactive oxygen species involved in initiation and progression of colon cancer [3,13]. In addition, sufficient intake of these phytochemicals in the diet may prevent cancer by decreasing the damage to DNA via oxidative stress, and also through enhanced DNA repair [9,10,15].

Berries such as blackberries, strawberries, raspberries and blueberries provide a rich, diverse and species-specific source of dietary antioxidants, especially the polyphenols, [16,12]. A recent study by Halvorsen *et al *stated that fruits from the *Rosaceae *family, including raspberries, were particularly rich in antioxidants [17]. Phytochemicals from different soft fruits, including raspberries, have been identified as effective inhibitors of oxidative DNA damage in immortalised human colon cells [18]. Other studies have shown that phytochemicals from berries, berry fractions and purified anthocyanins have antiproliferative effects on colon cancer cells *in vitro *[11,19,20]. There is also evidence that polyphenols present in fruit can prevent metastases and invasion of cancer cells *in vitro *[21,22].

Several studies have concentrated on the chemopreventive nature of specific polyphenols [23,24]. These studies involved single compounds used at high concentrations over an extended time period (up to 72 hr) [11,13,19,25]. Whilst this information may be useful to define the mechanisms involved in cancer development in humans [12], these studies do not allow for the possibility of additive or synergistic effects [10,15,12]. In addition, the experimental conditions are far from physiological. Phytochemicals differ widely in their bioavailability; some phytochemicals are poorly bioavailable [26] and/or are unstable under the conditions of the gastrointestinal tract (GIT) [27]. Therefore, studies carried out on pure phytochemicals [28] or berry extracts [11] are unrealistic unless the bioavailability of the phytochemicals has been well defined. In the present study, we have used an *in vitro *digestion procedure that mimics the physiochemical changes that occur in the upper GIT, particularly the stomach and small intestine, to produce a representative set of phytochemicals that could come into contact with the colonic epithelium *in situ*. The aim of this study is to examine the effects of this colon-available raspberry extract on a series of events that are biologically relevant to CRC, and which represent the major stages in colorectal carcinogenesis: initiation, promotion and metastasis. These *in vitro *studies use cell lines widely employed as models of colorectal cancer including HT29 for DNA damage, genotoxicity and cell cycle events, HT115 for invasion studies, and CACO-2 cells for barrier function studies.

## Methods

### Reagents

Dulbecco's Modified Eagle Medium (DMEM), Minimum Essential Medium (MEM), and foetal bovine serum (FBS) were obtained from Gibco Life Technologies Ltd (Paisley, Scotland, UK). Non-essential amino acids (NEAA), *l*-glutamine, penicillin/streptomycin, hydrogen peroxide, propionate, trypsin, and deoxycholic acid were obtained from Sigma-Aldrich Company Ltd (Dorset, England, UK).

### Raspberry extract

The study used Scottish raspberries (*Rubus idaeus *L. variety Glen Ample) purchased at full ripeness from farmers local to the Scottish Crop Research Institute. Polyphenol-rich fractions devoid of vitamin C, organic acids and carotenoids were obtained using an adaptation of a well-described method [29] published previously [30].

Raspberry extract was obtained from ripe berries by homogenising with solvent (2% (v/v) glacial acetic acid in acetonitrile), then removing the solvent by rotary evaporation. The remaining raspberry extract was subjected to an *in vitro *procedure that simulates the digestive process as described previously [27]. The procedure was adapted from the method outlined by Gil-Izquierdo *et al *[31]. The method consists of two sequential steps; an initial pepsin/HCl digestion to simulate gastric conditions followed by a digestion with bile salts/pancreatin to simulate small intestine conditions. The final raspberry extract was diluted in distilled water to a concentration of soluble phenols similar to that of the original raspberry extract obtained after rotary evaporation.

Briefly, the original raspberry extract (final volume 20 ml) was then adjusted to pH 1.7 with 5N HCl then pepsin (Sigma Chem. Co Ltd) was added at 315 units/ml and incubated at 37°C in a heated water bath for two hours with shaking at 100 rpm. 2 ml aliquots of the post-gastric digestion were removed and frozen. The remainder was placed in a 250 ml glass beaker and 4.5 ml of 4 mg/ml pancreatin, 25 mg/ml bile salts mixture added. A segment of cellulose dialysis tubing (molecular mass cut-off 12 kDa) containing sufficient NaHCO_3 _to neutralise the sample's titratable acidity was added and the beaker sealed with parafilm. After 2 hours incubation at 37°C, the solution outside the dialysis tubing was taken as the OUT sample representing material that would reach the colon, and the solution inside the dialysis tubing was taken as the IN sample representing the serum available material. 2 ml samples of the IN and OUT material were taken and frozen. The NaHCO_3 _diffused out of the dialysis tubing and the pH of the OUT sample reached neutrality within 30–45 mins.

The amount of 1 M NaHCO_3 _required to neutralise an 18 ml aliquot of the post gastric digest plus 4.5 ml bile pancreatin/salts was defined as the titratable acidity. The post gastric, IN and OUT samples were thawed when required, centrifuged at 13,200 rcf in a microfuge and the supernatants assayed for anthocyanin and phenol content.

The total anthocyanin concentration was estimated by a pH differential method [32]. The absorbance value was related to anthocyanin content using the molar extinction coefficient calculated in-house for cyanidin-3-*0*-glucoside (CyG). Phenol content was measured using a modified Folin-Ciocalteau method [33] with a standard curve of gallic acid. All results have been corrected for the presence of phenols in the pancreatin/bile salts mixture.

The colon-available raspberry extract (CARE) was acidified to 0.5 % (v/v) by slow addition of 10% formic acid. The soluble material was passed through C18 solid phase extraction columns (1,000 mg capacity, Phenomenex Ltd, Macclesfield, UK) which had been pre-equilibrated in ultra pure water (UPW) containing 0.25 % (v/v) formic acid (FA). After a wash with 2 volumes of FA/UPW, the bound material was eluted by the addition of 0.25% (v/v) FA in 25 % (v/v) acetonitrile. This afforded complete separation of total phenolics from the bile salts present in the CARE samples. Recovery of cyanidin-3-*0*-glucoside from the SPE procedure was around 90%. The fractions were then dried in a speed-vac (Thermo-Scientific Ltd, High Wycombe, UK) to suitable phenol concentrations for LC-MS.

### Liquid Chromatography-Mass Spectroscopy (LC-MS^n^)

Samples (containing 40 μg gallic acid equivalents (GAE) by Folin assay) were analyzed on a LCQ-DECA system, comprising Surveyor autosampler, pump and photo diode array detector (PDAD) and a Thermo-Finnigan mass spectrometer iontrap. Three discrete channels were scanned at 280 nm, 365 nm and 520 nm. Samples were eluted over a gradient of 5% acetonitrile (0.5% formic acid) to 30% acetonitrile (0.5% formic acid) on a C18 column (Synergi Hydro C18 with polar end capping, 4.6 mm × 150 mm, Phenomenex Ltd, Macclesfield, UK) over 60 mins at a rate of 400 μl/min. The LCQ-DECA LC-MS was fitted with an electrospray ionisation interface and analyzed the samples in positive and negative ion mode. There were 2 scan events; full scan analysis followed by data dependent MS/MS of most intense ions. The data-dependent MS/MS used collision energies (source voltage) of 45% in wideband activation mode. Representative chromatographs are presented.

### Tissue culture

Human colon cancer cells HT29 (adenocarcinoma), HT115 (carcinoma), CACO-2 (adenocarcinoma), and human lung tissue MRC5 cells were obtained from the European Collection of Cell Cultures (ECACC), Salisbury, UK. HT29 and HT115 cells were cultured as monolayers in Roux flasks in DMEM culture medium containing 10% and 15% FBS respectively, 2 mM *l-*glutamine and 100 U/l penicillin/streptomycin. HT115 cell culture medium was also supplemented with 1% NEAA. CACO-2 and MRC5 cells were cultured as monolayers in Roux flasks in MEM culture medium containing 10% FBS, 2 mM *l-*glutamine and 100U/l penicillin/streptomycin and 1% NEAA. Cells were cultured for 7 days (up to 75% confluence) at 37°C with 5% CO_2 _and 95% humidity. The growth medium was changed every 2 days. Cells were washed for 2 min with PBS and re-suspended by the addition of trypsin (0.25% trypsin-EDTA) at 37°C for 5 min. Cells were centrifuged at 1,200 rpm for 3 min, the trypsin decanted and cells re-suspended in the appropriate medium.

### COMET assay

The effect of CARE on colonocyte DNA damage using the well-established HT29 cell model [34] was determined. Flasks of HT29 cells were incubated with CARE at various concentrations (0, 3.125, 6.25, 12.5, 25, 50 μg/ml) for 24 hr prior to the cells being harvested for subsequent assay. The genotoxic potential of CARE was assessed by treating HT29 cells with extracts in the absence of hydrogen peroxide. The HT29 cells pre-incubated with CARE were treated (75 μM H_2_O_2 _or PBS) for 5 min on ice, prior to centrifugation at 1,200 rpm for 5 min. Positive (HT29 cells + 75 μM H_2_O_2_) and negative (HT29 cells + PBS) controls were included in all experiments, and these control cells were not exposed to CARE.

After discarding the supernatant, the cells were reconstituted in 85 μl of 0.85% low melting point agarose in PBS and maintained in a water bath at 40°C. This suspension was mixed with 1% normal agarose gels on frosted slides and coverslips were added. The slides were subjected to lysis buffer (2.5 M NaCl, 100 mM Na_2_EDTA, 10 mM TRIS HCl) for 1 hr at 4°C and placed in electrophoresis buffer for 20 min to allow the DNA to unwind before electrophoresis at 25 V 300 mA for 20 min. Slides were washed (3 × 5 min) in neutralisation buffer (0.4 M TRIS HCl, pH = 7.5) at 4°C. All slides were stained with ethidium bromide (20 μl of 2 μg/ml) prior to scoring. Images were analysed at 400 × magnification using a Nikon eclipse 600 epi-fluorescence microscope. The % Tail DNA was recorded using Komet 3.0 image analysis software (Kinetic Imaging Ltd, Liverpool, UK). 100 cells per slide were scored. The mean was calculated from 100 cells/gel (each sample in triplicate) and the experiment repeated independently 3 times.

### Trans-epithelial resistance assay

Cultured CACO-2 cells form atypical brush border membranes and tight junctions through enterocytic differentiation [35] and they provide an appropriate and frequently used model for the study of permeability, barrier function and transportation [36,37]. Tight junction integrity (number and complexity of strands) correlates with the electrical resistance of the barrier formed. CACO-2 cells were seeded into 6-well plates with Transwell inserts (0.1% rat tail collagen coated polyethyleneterapthalate membranes; BD Bioscience, Bedford, UK) at a seeding density of 2.5 × 10^5 ^cells per well. The growth medium was changed every other day (apical = 2.5 ml, basolateral = 1.5 ml) for 21 days. Cells were maintained at 37°C in an atmosphere of 5% CO_2 _and 95% humidity. From day 7 the integrity of the monolayer was evaluated by measuring the trans-epithelial resistance (TER) (expressed as Ωcm^2^) using an EVOM epithelial voltohmmeter (World Precision Instruments Ltd, Aston, UK). The inserts were ready for experimentation when the TER values had stabilised, usually after 20 days of growth. The TER of the CACO-2 cell monolayers was measured at 0, 24 and 48 hours after addition of CARE (0, 3.125, 6.25, 12.5, 25, 50 μg/ml) to the apical compartment. Positive (10 mM Propionate) and negative (250 μM deoxycholic acid) controls were included in all experiments. Baseline resistance was recorded in the range of 929–1040 Ωcm^2 ^at the start of each experiment. The treatments were carried out in triplicate and each experiment repeated 3 times independently.

### Matrigel invasion assay

This assay has been modified from a method described previously [38]. Invasion rates of HT115 cells are enhanced in the presence of MRC5 foetal lung cells, as the latter secrete hepatocyte growth factor, a strong chemo-attractant, which has been shown to increase cell scattering and motility in HT115 cells [39,40]. Briefly, 6-well Biocoat Matrigel invasion chamber inserts (BD Bioscience) were rehydrated with 2 ml serum-free culture medium (37°C) for 2 hr. In a separate plate, MRC5 cells in suspension were seeded into the basolateral chambers (4 × 10^5 ^cells/well) and incubated at 37°C for 2 hr. The media and any unattached cells in each well was carefully removed and replaced with 2 ml of DMEM containing 10% FBS. The rehydrated inserts were then transferred to the plates containing the MRC5 cells where the media from the inserts was removed and replaced with 2 ml HT115 cell suspension (2 × 10^5 ^cells in serum-free DMEM) in the presence or absence of CARE (0, 3.125, 6.25, 12.5, 25, 50 μg/ml). Inserts containing cells plus media were used as a control. Plates were incubated at 37°C for 24 hr. After incubation the media from both chambers was removed and the cells in both chambers fixed with 70% ethanol for 30 min prior to staining with haematoxylin. Using a cotton bud, the non-invasive cells were removed from half of the membrane of the upper chamber by "scrubbing". This was repeated for the invasive cells on the other side of the insert. The number of invasive and non-invasive cells was then counted in 5 random fields, and the percentage invasion calculated. The total number of cells was also estimated by adding the mean number of invasive and non-invasive cells counted on the inserts. Each treatment was carried out in duplicate and the experiment repeated independently 3 times.

### Cell viability and cell cycle analysis

HT29 cells were harvested and seeded at 3 × 10^5 ^cells per flask into 25 cm^3 ^flasks 48 hr prior to treatment, and maintained at 37°C with 5% CO_2 _and 95% humidity. Media was replaced with media containing CARE (0, 3.125, 6.25, 12.5, 25, 50 μg/ml) and the cells incubated for 24 hr. Cells were then washed with PBS and re-suspended by the addition of 1 ml trypsin EDTA and incubated at 37°C for 5 min. The trypsin was inactivated by the addition of 2 ml of serum-containing medium. A cell viability count was carried out using a haemocytometer and trypan blue dye (Sigma). The cells centrifuged at 1,200 rpm for 3 min in polypropylene tubes (Becton Dickinson). The supernatant was decanted and the pellet re-suspended in a mixture of ice cold PBS and 70% ethanol/30% PBS (200 μl and 2 ml respectively). Cells were incubated for 30 min on ice before centrifugation at 1,200 rpm for 3 min, and the supernatant carefully discarded. Cells were then re-suspended in 800 μl of ice cold PBS, 100 μl of RNase A (1 mg/ml) (Sigma Chemicals), and 100 μl of propidium iodide (400 μg/ml) (Sigma Chemicals) before being incubated at 37°C for 30 min prior to analysis. Samples were processed on a FACSCalibur flow cytometer (Becton Dickinson) equipped with laser (excitation wave length = 488 nm). The fluorescence intensity of propidium iodide was collected at 585 nm (designated FL2 on FACSCalibur), using CellQuest Software (Becton Dickinson). In total, 10,000 events were captured. The intensities were analysed subsequently for DNA content using WinMDI Version 2.8 (Trotter Institute, JScripts, CA). Each experiment was completed in duplicate and the data set is the mean of 3 independent experiments.

### Statistical analysis

The mean of each data set was used for statistical analysis for each experiment. Analysis of variance was applied to test for significant differences between means by ANOVA and Dunnett T-test where significance was accepted at *p *< 0.05. These were carried out in SPSS (version 11.5) for Windows.

## Results

### Liquid Chromatography-Mass Spectroscopy (LC-MS^n^)

**Figure 1:**
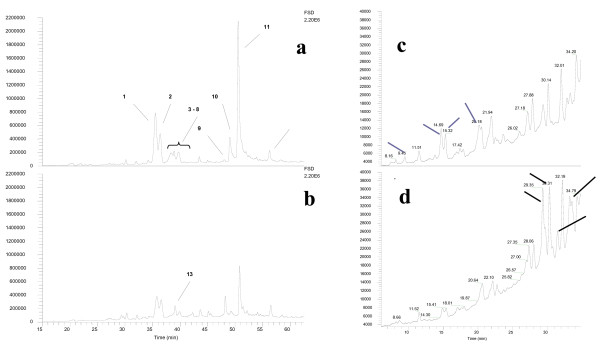
MS trace of berry extract and CARE. Comparison of undigested raspberry extract **(a) **and CARE **(b) **samples by LC-MS/MS, peaks 1 – 12 have been characterised (Table 1). Expanded area of LC trace of whole raspberry extract **(c) **and CARE **(d)**. Arrows in c denote peaks that are reduced by *in vitro *digestion, arrows in d denote peaks that increase in abundance or are only present in the digested sample.

The original raspberry extract had a similar polyphenol composition to previous reports [41,42] with an anthocyanin content of 15.9 μg/ml compared to 100 μg/ml for total phenols. The HPLC profile of the raspberry extract was similar to previous reports [43,27,30,45] and was mainly composed of anthocyanins (peaks 1–8; Table 1) and ellagitannins (peaks 9–11; Table 1) with a number of other minor components (Figure 1a). The anthocyanins were detected by their absorption at 520 nm and their structures confirmed by mass spectrometry (Table 1). The most abundant anthocyanin was cyanidin-3-*O*-sophoroside (peak 1), peak 2 was composed of cyanidin-3-*O*-(2^G^)-glucosylrutinoside and peaks 3–8 were not completely separated but were composed of cyanidin-3-*O*-glucoside, pelargonidin-3-*O*-sophoroside, cyanidin-3-*O*-rutinoside, pelargonidin-3-*O*-(2^G^)-glucosylrutinoside. Smaller amounts of pelargonidin-3-*O*-glucoside and pelargonidin-3-*O*-rutinoside eluted later and could be confirmed by searching the MS data at their relevant masses.

**Table 1:** Characterisation and identification of CARE by MS

**Peak No.**	**T**_R_	***m/z***^+^	**MS**^2^	**Putative Identity**	% Recovery*
1	35.52	611.1	**287.2**	Cyanidin-3-*O*-sophoroside	33.8 ± 1.6
2	36.37	757.3	**287.2**, 611.1	Cyanidin-3-*O*-glucosylrutinioside	48.9 ± 2.6
3	38.34	449.1	**287.2**	Cyanidin-3-*O*-glucoside	15.5 ± 3.3
4	38.86	595.1	**271.2**, 595.1	Pelargonidin-3-*O*-sophoroside	32.7 ± 3.1
5	39.59	595.1	**287.2**, 449.1	Cyanidin-3-*O*-rutinoside	27.1 ± 3.0
6	38.87	741.1	**271.1**, 595.1	Pelargonidin-3-*O*-glucosylrutinoside	50.0 ± 2.6
7	42.43	433.1	**271.1**	Pelargonidin-3-*O*-glucoside	33.0 ± 1.9
8	43.38	579.1	**271.1**, 433.0	Pelargonidin-3-*O*-rutinoside	52.1 ± 3.2
		***m/s***^-^			
9	48.01	1567.1	*multiple*	Sanguiin H6	613.6 ± 14.5
10	49.03	1401.2	*multiple*	Lambertinian C/Sanguiin H10	16.3 ± 2.0
11	50.54	1869.0	*multiple*	Sanguiin H10	27.6 ± 2.2
12	56.25	N/A	N/A	Hydroxycinnamic acid derivative	61.7 ± 4.3**

* The % recovery based on the peak area of the mass spectrometer response for each m/z. The value is the average of triplicate samples ± standard errors.

** The recovery of peak 12 was based on PDA peak size.

Peaks 9–11 absorbed well in the UV and gave maxima around 285 nm. Peak 11 yielded one predominant mass ion at *m/z *1869 in negative mode (Table 1) with smaller peaks at 1567, 1235, 933, and 633, which may arise from in-source fragmentation of the *m/z *1869 ion. Indeed, the *m/z *1869 ion gave MS^2 ^ions at *m/z *1567, 1235, 1103, 933, 897 and 633. The mass ion and the fragment ions were essentially identical to the pattern obtained for the ellagitannin, Sanguiin H6 [46], which has previously been identified in near-identical raspberry extracts [27,30,45]. Peak 9 yielded a dominant ion at *m/z *1567 in negative mode (Table 1) which has been assigned to Sanguiin H10 [46], which has a similar structure to Sanguiin H6 but lacks one hexahdroxydiphenoyl (HHDP) unit. Peak 10 gave an MS spectrum similar to that of peak 11 except it also contained an appreciable signal at *m/z *1401 (Table 1), which has been assigned to Lambertianin C [46]. Therefore, Peak 11 was composed of a mixture of Lambertianin C and Sanguiin H6 similar to that described in previous work [27,30].

The CARE contained the same major components as the original raspberry extract (Figure 1b; Table 1) but with generally depleted levels (e.g. compare the full scale deflectance (FSD) of Figure 1a and 1b). Peak 13 increased in apparent abundance but largely because it was uncovered as the levels of the anthocyanins in peaks 3–8 dropped. This compound gave a PDAD spectrum with a maximum around 325 nm and a shoulder around 280 nm, which suggests a hydroxycinnamic acid derivative [47] but gives no clear *M/Z *or MS^2 ^signals in positive or negative mode. A number of other peaks were either reduced (Figure 1c) by *in vitro *digestion or were uncovered or generated (Figure 1d) by this procedure. *In vitro *digestion reduced the total phenolic content and the total anthocyanin content of the CARE to 72.3 % and 83.8 % respectively compared to the original extract. These recoveries appear to be considerably higher than the recovery of individual polyphenol components (which ranged from 13 - >60 %). However, certain components (e.g. peaks 9) increased in abundance and other degradation products of the main components may also contribute to the total phenol content (by Folins) but may be unidentified by LC-MS.

### COMET assay

**Figure 2:**
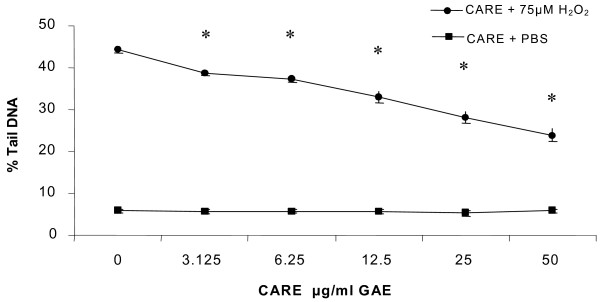
Genotoxic and anti-genotoxic effects of CARE (24 hr incubation) at different concentrations on DNA damage in HT29 cells, *n *= 3, mean ± SD, *p < 0.001. Hydrogen peroxide challenge 75 μM.

There was no significant genotoxic activity observed at any of the concentrations of CARE (Figure 2) used for pre-incubation (24 hr) as DNA damage was similar to that seen in untreated controls (5.75 ± 0.48 % Tail DNA,). A significant anti-genotoxic effect (*p *< 0.01) was observed for HT29 cells challenged with hydrogen peroxide (75 μM for 5 min) when pre-treated with CARE in a dose-dependant manner (Figure 2) at all concentrations tested. At the highest concentration of CARE, 50 μg/ml GAE, there was maximum reduction in DNA damage of approximately 50% (23.87 ± 1.54 %) verses the positive control (44.22 ± 0.62 % Tail DNA). At the lowest concentration of CARE (3.125 μg/ml GAE) there was a significant decrease in % tail DNA of approximately 15% (38.5 ± 0.55 % tail DNA) verses the positive control.

### Trans-epithelial resistance assay

The assessment of barrier function was made by recording changes in transepithelial resistance over a 48 hour time period after the addition of CARE, with measurements taken at T_0_, T_24 _and T_48_. The addition of CARE over a range of concentrations (0 – 50 μg/ml GAE) had no significant effect on barrier function of the CACO-2 monolayer (data not shown).

### Cell viability and cell cycle analysis

**Figure 3:**
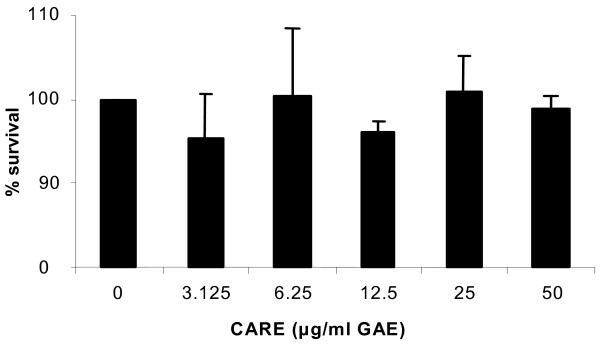
Cytotoxic effect of CARE on HT29 cells (*n *= 3). No significant cytotoxic effect was observed at any of the concentrations used (0, 3.125, 6.25, 12.5, 25, 50 μg/ml).

The dose range of CARE tested had no significant effect on cytotoxicity, as seen in the cell viability data (Figure 3). The effect of CARE treatment on HT29 cell proliferation was also evaluated by measuring cell cycle distribution. The incubation of HT29 cells for 24 hr with 50 μg/ml GAE CARE caused an approximate 10% decrease in the proportion of cells in the G_0_/G_1 _phase of cell cycle, relative to control cells (*p *< 0.05). There were corresponding increases in cells in the S and G_2_/M phases but these changes were not significant. At all other concentrations tested no significant changes were observed and the cell cycle profile closely resembled that of the control cells.

### Matrigel invasion assay

The effect of different concentrations of CARE on Matrigel invasion by HT115 cells is shown in Figure 5. At the lowest concentration used (3.125 μg/ml GAE), the addition of CARE had no significant effect on invasion rates compared to the control (0 μg/ml GAE, 11.25 ± 1.88 % invasion). All other concentrations of CARE (6.25, 12.5, 25 & 50 μg/ml GAE) significantly inhibited the invasion of HT115 cells (7.16 ± 2.11, 3.95 ± 1.34, 2.04 ± 0.87 and 0.82 ± 0.46 % invasion respectively). A dose-dependent decrease in invasion was observed with increasing CARE concentration (r^2 ^= 0.99). At the lowest significant concentration of CARE (6.25 μg/ml GAE), a decrease in invasion of approximately 40% was observed, whilst at the highest concentration (50 μg/ml GAE) the decrease in invasion was approximately 90%.

## Discussion

The use of crude homogenates of fruit and vegetables in *in vitro *studies in colon cells may overestimate the biological activity since no account is taken of digestion and absorption on the phytochemical composition. The simulated digestion used here is not a true representation of human digestion. For example, the length of digestion is arbitrarily chosen from average transit figures and it cannot replicate the effects of brush border enzyme systems [48]. It also cannot replicate the effect of microflora in the stomach and small intestine [49]. However it mimics key physiochemical changes of the digestive process to provide an extract similar in composition to that found to be colon-available *in vivo *[27].

*In vitro *digestion of the raspberry extract produced the colon-available raspberry extract (CARE) which retained a considerable portion of the original anthocyanin content (~50%) and the total phenol content (~70%). These figures are in line with previous studies on the stability of raspberry anthocyanins [27] and are similar to other *in vitro *digestion studies on chokeberry [50] and pomegranate [51].

However, the composition of the individual polyphenolic components in raspberry is dynamic. For example, certain anthocyanins are less stable than others. The general pattern is similar to previous reports [27] with cyanidin-3-*O*-glucoside being the least stable anthocyanin. The low recovery may be due to sacrificial oxidation in the raspberry mixture as cyanidin-3-*O*-glucoside has a higher antioxidant capacity than other anthocyanins [52] and may be preferentially oxidised.

That ellagitannins are also unstable to the conditions of gastrointestinal digestion has been well documented [53,54]. Nevertheless, ~20% of the original content of lambertianin C and Sanguiin H10 survived the *in vitro *digestion and the 6-fold increase in recovery of Sanguiin H6 would also contribute to a substantial dose of ellagitannins reaching the colon.

The survival of polyphenols into the colon is well documented. Anthocyanins have been detected in faecal contents [55] and throughout the gastrointestinal tract in pigs [56]. A large portion of ingested anthocyanins and other polyphenols pass intact into the colon fraction [57-60] and may be degraded by fermentation into smaller phenolic compounds by gut microflora and re-absorbed to contribute to the serum antioxidant capacity.

The increased recovery of Sanguiin H6 is almost certainly due to the breakdown of Sanguiin H10 through the loss of a HHDP unit [61], which spontaneously re-arranges to ellagic acid (EA). EA has been shown to be an effective anti-cancer agent [62]. The pomegranate ellagitannin, punicalagin degraded to produce EA in the mildly alkaline media of cultured CACO-2 cells [63] and raspberry-derived Sanguiin H10 degrades to EA in similar media of HeLa cervical cancer cells [45]. However, free EA was not detected after gastrointestinal digestion. Initial experiments have suggested that EA may have been bound to proteins in the pancreatin/bile salts mixture and escaped recovery by solid phase extraction (results not shown). However, EA bound in this manner may be released *in vivo *as the proteins are degraded or as the pH drops in the fermentative caecum [58]. Indeed, urinary excretion of urolithin-type degradation products of ellagic acid was estimated at 14 % of ellagitannin intake from raspberries [60], which confirms that ellagitannins (and therefore ellagic acid) are retained in the gastrointestinal tract and biotransformed by the colonic microflora.

Cells in the gastrointestinal tract, particularly the colon, are susceptible to develop mutations due to dietary carcinogens present in the gut, which are in direct contact with cells of the colon (and may access the blood stream and cause cell mutations) [64]. These carcinogens trigger events such as free radical formation and production of reactive oxygen species, which can cause DNA damage, and can initiate the cancer process. Conversely, the gut contents may contain food-derived anti-cancer agents [65]. A dose response effect was observed for anti-genotoxicity with increasing CARE concentration in HT29 human colon cells challenged with H_2_O_2_. The reduction (approximately 50% at the highest concentration used) in H_2_O_2_-induced DNA damage indicates increased cellular capabilities to protect against damage. Cyanidin-3-glucoside, a common berry anthocyanin, pre-incubated for 4 hours at 50 μM with human colon epithelial cells resulted in almost 40% reduction in DNA damage caused by a H_2_O_2_insult at 200 μM [18]. This concentration is equivalent to 22.5 μg/ml, which is in the same effective range as the CARE samples used in this study. Preincubation with the aglycone cyanidin (100 μM for 30 min) caused an approximate 10% decrease in H_2_O_2_-induced DNA damage in HT29 cells when subjected to oxidative insult (75 μM H_2_O_2_) [20]. Therefore, CARE has a similar protective effect against DNA damage as other phytochemicals at similar concentrations. However, whilst the use of isolated and purified phytochemicals is relevant to determine specific effects, we maintain that use of a whole extract, subjected to *in vitro *digestion, is more physiologically-relevant to the situation after ingestion of berries and allows the detection of possible synergistic effects of phytochemicals.

Cell proliferation is important in the development of colon cancer from the initiation stage. As the cells continue to divide, they acquire more defects in key genes allowing the cell to continue growing [7]. Cell proliferation in normal cells is regulated at the cell cycle checkpoints G_1_/S and G_2_/M, at which time correct DNA synthesis and chromosomal segregation is maintained. However in cancerous cells, particularly in colon cancer, these regulatory mechanisms are defective, and cells undergo growth and division unchecked. Therefore, we investigated the effect of CARE on proliferation of colon cancer cells by measuring barrier function and cell cycle profile. There was no significant increase or decrease in barrier function of CACO-2 cells at any concentrations of CARE used, suggesting that the addition of digested raspberry extract had no effect on epithelial integrity. However, a significant decrease in HT29 cells in the G_0_/G_1 _phase of the cell cycle was observed at the highest concentration used (50 μg/ml GAE), causing a slight, but not statistically significant increase in cells accumulating in the S and G_2_/M phases. This suggests that although cells treated with CARE have circumvented the normal cell cycle control at G_1_/S, they are unable to complete the cell cycle, indicating that CARE could restrict cell proliferation *in vitro*. Anthocyanin-rich extracts from chokeberries inhibited growth and cell cycle progression of HT29 cells [66]. Approximately 60% growth inhibition was observed after a 24 hr pre-incubation with 50 μg/ml extract, and the cell cycle was halted at the G_1_/S and G_2_/M checkpoints [66]. Therefore, CARE reduced cell cycle progression and prevented cell proliferation at similar concentrations to other berry extracts. There is evidence that there is considerable potential for antiproliferative synergy between polyphenolic components. Seeram *et al*. observed approximately 30% inhibition in proliferation in a human metastatic colon cancer cell line (SW620) when pre-treated with total cranberry extract (200 μg/ml for 48 hr) [19]. At similar concentrations, isolated cranberry anthocyanins caused an approximate 10% decrease in cell proliferation, indicating an additive or synergistic antiproliferative effect resulting from the combination of phytochemicals present in the extract. Similar synergistic effects on apoptotic cell death in human colon HT29 and HT116 cells were noted for whole pomegranate extracts (100 μg/ml for 24 hours) over isolated pomegranate ellagitannins [67].

The final important stage in cancer development is the spread of cancer to other tissues. CARE (at 6.25 – 50 μg/ml GAE) significantly reduced the invasiveness of HT115 colon cancer cells *in vitro*. At the highest concentration of CARE, there was almost a 95% reduction in cell invasiveness and this reduction was directly related to CARE dose. In other studies, Kim *et al*. demonstrated a 75% inhibition of invasion in MCF-7 breast cancer cells using pomegranate seed oil at 10 μg/ml [68]. Pomegranate seed oil contains various phytochemicals including β-sitosterol and anthocyanins, which previous studies have shown are important in regulating various stages of carcinogenesis [13,18]. However, little is known about the stability of these components to gastrointestinal digestion. Epigallocatechin-3-gallate (EGCG), which is isolated from green tea, can inhibit invasion of various carcinomas, including pancreatic carcinoma cells [69], and human biliary tract carcinoma cells [70] at concentrations of 100 μg/ml. The invasive capacity of pancreatic cell lines, PANC-1, MIA PaCa-2 and BxPC-3, was reduced after pre-treatment with 100 μg/ml EGCG for 2 hours [69]. It is notable that CARE was effective at a similar concentration and timescale, even though direct comparison between different cell lines and different extracts is difficult.

Whilst the bioavailability of different phytochemicals remains relatively unexplored, many polyphenols have poor *in vivo *serum bioavailability [71,72,26,65]. In particular, the major polyphenol components of raspberry, anthocyanins and ellagitannins, are generally considered to have poor serum bioavailability [67,52,73]. However, cancers of the alimentary canal are unique in that the abnormal cells can be in direct contact with food-derived potential anti-cancer agents present in the intestinal lumen. Also, unabsorbed phytochemicals may have direct chemopreventive activity on cells predisposed to develop mutations in the gastrointestinal tract, without accessing the blood circulation system, through prevention of lipid/protein oxidation [65,74]. New polyphenol compounds were formed during the digestive process, which were not present in the original raspberry extract. Further work is required to discern the nature of these components although it is probable that they are breakdown products formed from the major components. Although present in small amounts, these components may have significant effects (alone or in concert) that would not be observed when using a purified compound or a non-digested extract.

## Conclusion

A considerable weight of evidence has been gathered that suggests that consumption of fruit and vegetables is advantageous to our health and may help to prevent chronic diseases such as cancer [1-3]. The data obtained in our *in vitro *study supports this view and provides insight into the possible stages at which raspberry phytochemicals may act to halt the progression of cancer. We have demonstrated that CARE can inhibit *in vitro *models of key stages in colorectal cancer development, namely initiation, promotion and invasiveness. Further insights into the anti-cancer effects of CARE may require the use of a suitable animal model.

## Competing interests

The author(s) declare that they have no competing interests.

## Authors' contributions

EMC carried out the TER assay and drafted the manuscript. GP carried out the Comet assay and the matrigel invasion assay. MJMC performed cell viability and cell cycle analysis. GJMD conceived the study, carried out extract preparation and characterization by LC-MS and helped draft the manuscript. CIRG conceived and designed the study, provided data analysis and helped draft the manuscript. DS, IR conceived the study and critically reviewed the manuscript.

**Figure 4:**
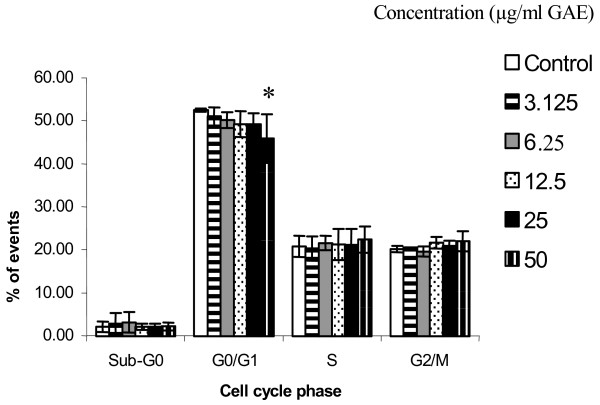
Analysis of cell cycle distribution of HT29 cells incubated with CARE, *n *= 3, mean ± SD, *p = 0.024.

**Figure 5:**
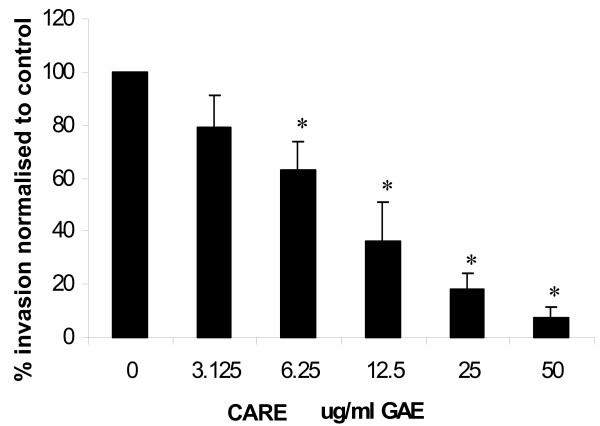
Inhibitory effect of CARE at different concentrations on HT115 cell invasion rates in a dose dependant manner, *n *= 3, mean ± SD, *p < 0.05. Values expressed as % cell invasion normalised to control (0 μg/ml).

## References

[B1] Anon (2002). Joint WHO/FAO Expert Consultation on Diet, Nutrition and the Prevention of Chronic Diseases. WHO technical report series, Geneva, Switzerland.

[B2] Liu RH (2003). Health benefits of fruit and vegetables are from additive and synergistic combinations of phytochemicals. Am J Clinical Nutrition.

[B3] Duthie S, Jenkinson A, Crozier A, Mullen W, Pirie L, Kyle J, Yap L, Christen P, Duthie G (2006). The effects of cranberry juice consumption on antioxidant status and biomarkers relating to heart disease and cancer in healthy human volunteers. Eur J Nutr.

[B4] Johnson I (2004). New approaches to the role of diet in the prevention of cancers of the alimentary tract. Mutat Res.

[B5] Bruce WR, Giacca A, Medline A (2000). Possible Mechanisms Relating Diet and Risk of Colon Cancer. Cancer Epidemiol Biomarkers Prev.

[B6] Goldstein N (2006). Serrated pathway and APC (conventional)-type colorectal polyps: molecular-morphologic correlations, genetic pathways, and implications for classification. Am J Clin Pathol.

[B7] Arnold C, Goel A, Blum H, Boland C (2005). Molecular pathogenesis of colorectal cancer: implications for molecular diagnosis. Cancer.

[B8] Halliwell B (1996). Free radicals, proteins and DNA: oxidative damage versus redox regulation. Biochem Soc Trans.

[B9] Liu M, Li X, Weber C, Lee C, Brown J, Liu R (2002). Antioxidant and antiproliferative activities of raspberries. J Agric Food Chem.

[B10] Sun J, Chu Y, Wu X, Liu R (2002). Antioxidant and antiproliferative activities of common fruits. J Agric Food Chem.

[B11] Olsson M, Gustavsson K, Andersson S, Nilsson A, Duan R (2004). Inhibition of cancer cell proliferation in vitro by fruit and berry extracts and correlations with antioxidant levels. J Agric Food Chem.

[B12] Lu H, Li J, Zhang D, Stoner G, Huang C (2006). Molecular mechanisms involved in chemoprevention of black raspberry extracts: from transcription factors to their target genes. Nutr Cancer.

[B13] Han C, Ding H, Casto B, Stoner G, D'Ambrosio S (2005). Inhibition of the growth of premalignant and malignant human oral cell lines by extracts and components of black raspberries. Nutr Cancer.

[B14] Liu RH (2004). Potential Synergy of Phytochemicals in Cancer Prevention: Mechanism of Action. J Nutr.

[B15] Freese R (2006). Markers of oxidative DNA damage in human interventions with fruit and berries. Nutr Cancer.

[B16] Machiex JFA, Billot J (1990). Fruit Phenolics.

[B17] Halvorsen BL, Holte K, Myhrstad MCW, Barikmo I, Hvattum E, Remberg SF, Wold A-B, Haffner K, Baugerod H, Andersen LF, Moskaug O, Jacobs DR, Blomhoff R (2002). A Systematic Screening of Total Antioxidants in Dietary Plants. J Nutr.

[B18] Duthie S, Gardner P, Morrice P, Wood S, Pirie L, Bestwick C, Milne L, Duthie G (2004). DNA stability and lipid peroxidation in vitamin E-deficient rats in vivo and colon cells in vitro. Modulation by the dietary anthocyanin, cyanidin-3-glycoside. Eur J Nutr.

[B19] Seeram N, Adams L, Hardy M, Heber D (2004). Total cranberry extract versus its phytochemical constituents: antiproliferative and synergistic effects against human tumor cell lines. J Agric Food Chem.

[B20] Glei M, Matuschek M, Steiner C, Bohm V, Persin C, Pool-Zobel B (2003). Initial in vitro toxicity testing of functional foods rich in catechins and anthocyanins in human cells. Toxicol In Vitro.

[B21] Lansky E, Jiang W, Mo H, Bravo L, Froom P, Yu W, Harris N, Neeman I, Campbell M (2005). Possible synergistic prostate cancer suppression by anatomically discrete pomegranate fractions. Invest New Drugs.

[B22] Liu J, Chen S, Lin C, Tsai S, Liang Y (2001). Inhibition of melanoma growth and metastasis by combination with (-)-epigallocatechin-3-gallate and dacarbazine in mice. J Cell Biochem.

[B23] Heber D (2004). Phytochemicals beyond Antioxidation. J Nutr.

[B24] Mouat M, Kolli K, Orlando R, Hargrove J, Grider A (2005). The effects of quercetin on SW480 human colon carcinoma cells: a proteomic study. Nutr J.

[B25] Zhao C, Giusti M, Malik M, Moyer M, Magnuson B (2004). Effects of commercial anthocyanin-rich extracts on colonic cancer and nontumorigenic colonic cell growth. J Agric Food Chem.

[B26] Manach C, Scalbert A, Morand C, Remesy C, Jimenez L (2004). Polyphenols: food sources and bioavailability. Am J Clinical Nutrition.

[B27] McDougall G, Dobson P, Smith P, Blake A, Stewart D (2005). Assessing potential bioavailability of raspberry anthocyanins using an in vitro digestion system. J Agric Food Chem.

[B28] Shih P, Yeh C, Yen G (2005). Effects of anthocyanidin on the inhibition of proliferation and induction of apoptosis in human gastric adenocarcinoma cells. Food Chem Toxicol.

[B29] Rommel A, RE W (1993). Composition of flavanols in red raspberry juice as influenced by cultivar, processing and environmental factors. J Agric Food Chem.

[B30] McDougall G, Shpiro F, Dobson P, Smith P, Blake A, Stewart D (2005). Different polyphenolic components of soft fruits inhibit alpha-amylase and alpha-glucosidase. J Agric Food Chem.

[B31] Gil-Izquierdo A, Gil M, Ferreres F, Tomas-Barberan F (2001). In vitro availability of flavonoids and other phenolics in orange juice. J Agric Food Chem.

[B32] Riberreau-Gayon P (1965). Determination of anthocyanins in red wine. Dull Soc Chim Fr.

[B33] Singleton VRJ (1965). Colirimetry of total phenolics with phosphomolybdic-phosphotungstic acid reagents. Amer j Enol Viticult.

[B34] Gill C, Boyd A, McDermott E, McCann M, Servili M, Selvaggini R, Taticchi A, Esposto S, Montedoro G, McGlynn H, Rowland I (2005). Potential anti-cancer effects of virgin olive oil phenols on colorectal carcinogenesis models in vitro. Int J Cancer.

[B35] Pinto MR-LS, Appay MD, Kedinger M, Triadou N, Dussaulx E, Lacroix B, Simon-Assman P, Haffen K, Fogh J, Zwwibaum A (1983). Enterocyte-like differentiation and polarization of the human colon carcinoma cell line CaCo-2 in culture. Biol Cell.

[B36] Nowak P, Blaheta R, Schuller A, Cinatl J, Wimmer-Greinecker G, Moritz A, Scholz M (2004). The Na+/H+ exchange inhibitor HOE642 prevents stress-induced epithelial barrier dysfunction. Int J Mol Med.

[B37] Diebel L, Liberati D, Dulchavsky S, Diglio C, Brown W (2003). Enterocyte apoptosis and barrier function are modulated by SIgA after exposure to bacteria and hypoxia/reoxygenation. Surgery.

[B38] Parr C, Hiscox S, Nakamura T, Matsumoto K, Jiang W (2000). Nk4, a new HGF/SF variant, is an antagonist to the influence of HGF/SF on the motility and invasion of colon cancer cells. Int J Cancer.

[B39] Jiang W, Puntis M, Hallett M (1993). Monocyte-conditioned media possess a novel factor which increases motility of cancer cells. Int J Cancer.

[B40] Hiscox S, Jiang W (1999). Association of the HGF/SF receptor, c-met, with the cell-surface adhesion molecule, E-cadherin, and catenins in human tumor cells. Biochem Biophys Res Commun.

[B41] Kahkonen M, Hopia A, Heinonen M (2001). Berry phenolics and their antioxidant activity. J Agric Food Chem.

[B42] Deighton NBR, Finn C, Davies HV (2000). Antioxidant properties of domesticated and wild *Rubus *species. J Sci Food Agric.

[B43] Mullen W, McGinn J, Lean M, MacLean M, Gardner P, Duthie G, Yokota T, Crozier A (2002). Ellagitannins, flavonoids, and other phenolics in red raspberries and their contribution to antioxidant capacity and vasorelaxation properties. J Agric Food Chem.

[B44] Beekwilder J, Jonker H, Meesters P, Hall R, van der Meer I, Ric de Vos C (2005). Antioxidants in raspberry: on-line analysis links antioxidant activity to a diversity of individual metabolites. J Agric Food Chem.

[B45] Ross H, McDougall G, Stewart D (2006). Antiproliferative activity is predominantly associated with ellagitannins in raspberry extracts. Phytochemistry.

[B46] Mullen W, Yokota T, Lean M, Crozier A (2003). Analysis of ellagitannins and conjugates of ellagic acid and quercetin in raspberry fruits by LC-MSn. Phytochemistry.

[B47] Maatta K, Kamal-Eldin A, Torronen A (2003). High-performance liquid chromatography (HPLC) analysis of phenolic compounds in berries with diode array and electrospray ionization mass spectrometric (MS) detection: ribes species. J Agric Food Chem.

[B48] Van Beers E, Buller H, Grand R, Einerhand A, Dekker J (1995). Intestinal brush border glycohydrolases: structure, function, and development. Crit Rev Biochem Mol Biol.

[B49] Guarner F, Malagelada J (2003). Gut flora in health and disease. Lancet.

[B50] Bermudez-Soto M-TT-B FA, Carcia-Conesa M-T (2006). Stability of polyphenols in chokeberry (*Aronia melanocarpa*) subjected to in vitro gastric and pancreatic digestion. Food Chem.

[B51] Perez-Vicente A, Gil-Izquierdo A, Garcia-Viguera C (2002). In vitro gastrointestinal digestion study of pomegranate juice phenolic compounds, anthocyanins, and vitamin C. J Agric Food Chem.

[B52] McGhie T, Ainge G, Barnett L, Cooney J, Jensen D (2003). Anthocyanin glycosides from berry fruit are absorbed and excreted unmetabolized by both humans and rats. J Agric Food Chem.

[B53] Daniel E, Ratnayake S, Kinstle T, Stoner G (1991). The effects of pH and rat intestinal contents on the liberation of ellagic acid from purified and crude ellagitannins. J Nat Prod.

[B54] Clifford MSA (2000). Ellagitannins – nature, occurrence and dietary burden. J Sci Food Agric.

[B55] He J, Magnuson B, Giusti M (2005). Analysis of anthocyanins in rat intestinal contents–impact of anthocyanin chemical structure on fecal excretion. J Agric Food Chem.

[B56] Wu X, Pittman H, Prior R (2006). Fate of anthocyanins and antioxidant capacity in contents of the gastrointestinal tract of weanling pigs following black raspberry consumption. J Agric Food Chem.

[B57] Rechner A, Kuhnle G, Hu H, Roedig-Penman A, van den Braak M, Moore K, Rice-Evans C (2002). The metabolism of dietary polyphenols and the relevance to circulating levels of conjugated metabolites. Free Radic Res.

[B58] Gonthier M-P, Cheynier V, Donovan JL, Manach C, Morand C, Mila I, Lapierre C, Remesy C, Scalbert A (2003). Microbial Aromatic Acid Metabolites Formed in the Gut Account for a Major Fraction of the Polyphenols Excreted in Urine of Rats Fed Red Wine Polyphenols. J Nutr.

[B59] Aura A, Martin-Lopez P, O'Leary K, Williamson G, Oksman-Caldentey K, Poutanen K, Santos-Buelga C (2005). In vitro metabolism ofanthocyanins by human gut microflora. Eur J Nutr.

[B60] Cerda B, Tomas-Barberan F, Espin J (2005). Metabolism of antioxidant and chemopreventive ellagitannins from strawberries, raspberries, walnuts, and oak-aged wine in humans: identification of biomarkers and individual variability. J Agric Food Chem.

[B61] Porter L (1993). Methods in Plant Biochemistry.

[B62] Losso J, Bansode R, Trappey A, Bawadi H, Truax R (2004). In vitro anti-proliferative activities of ellagic acid. J Nutr Biochem.

[B63] Larrosa M, Tomas-Barberan F, Espin J (2006). The dietary hydrolysable tannin punicalagin releases ellagic acid that induces apoptosis in human colon adenocarcinoma Caco-2 cells by using the mitochondrial pathway. J Nutr Biochem.

[B64] Nakagama H, Nakanishi M, Ochiai M (2005). Modeling human colon cancer in rodents using a food-borne carcinogen, PhIP. Cancer Sci.

[B65] Lala G, Malik M, Zhao C, He J, Kwon Y, Giusti M, Magnuson B (2006). Anthocyanin-rich extracts inhibit multiple biomarkers of colon cancer in rats. Nutr Cancer.

[B66] Malik M, Zhao C, Schoene N, Guisti M, Moyer M, Magnuson B (2003). Anthocyanin-rich extract from Aronia meloncarpa E induces a cell cycle block in colon cancer but not normal colonic cells. Nutr Cancer.

[B67] Seeram N, Adams L, Henning S, Niu Y, Zhang Y, Nair M, Heber D (2005). In vitro antiproliferative, apoptotic and antioxidant activities of punicalagin, ellagic acid and a total pomegranate tannin extract are enhanced in combination with other polyphenols as found in pomegranate juice. J Nutr Biochem.

[B68] Kim N, Mehta R, Yu W, Neeman I, Livney T, Amichay A, Poirier D, Nicholls P, Kirby A, Jiang W, Mansel R, Ramachandran C, Rabi T, Kaplan B, Lansky E (2002). Chemopreventive and adjuvant therapeutic potential of pomegranate (Punica granatum) for human breast cancer. Breast Cancer Res Treat.

[B69] Takada M, Nakamura Y, Koizumi T, Toyama H, Kamigaki T, Suzuki Y, Takeyama Y, Kuroda Y (2002). Suppression of human pancreatic carcinoma cell growth and invasion by epigallocatechin-3-gallate. Pancreas.

[B70] Singh A, Seth P, Anthony P, Husain M, Madhavan S, Mukhtar, Maheshwari R (2002). Green tea constituent epigallocatechin-3-gallate inhibits angiogenic differentiation of human endothelial cells. Arch Biochem Biophys.

[B71] Scalbert A, Williamson G (2000). Dietary Intake and Bioavailability of Polyphenols. J Nutr.

[B72] Williamson G, Manach C (2005). Bioavailability and bioefficacy of polyphenols in humans. II. Review of 93 intervention studies. Am J Clinical Nutrition.

[B73] Stoner GD, Sardo C, Apseloff G, Mullet D, Wargo W, Pound V, Singh A, Sanders J, Aziz R, Casto B, Sun X (2005). Pharmacokinetics of Anthocyanins and Ellagic Acid in Healthy Volunteers Fed Freeze-Dried Black Raspberries Daily for 7 Days. J Clin Pharmacol.

[B74] He J, Magnuson B, Lala G, Tian Q, Schwartz S, Giusti M (2006). Intact anthocyanins and metabolites in rat urine and plasma after 3 months of anthocyanin supplementation. Nutr Cancer.

